# Identification and Validation of the Signatures of Infiltrating Immune Cells in the Eutopic Endometrium Endometria of Women With Endometriosis

**DOI:** 10.3389/fimmu.2021.671201

**Published:** 2021-09-03

**Authors:** Xiang-Guang Wu, Jin-Jiao Chen, Hui-Ling Zhou, Yu Wu, Fei Lin, Jing Shi, Hong-Zhen Wu, Hai-Qun Xiao, Wei Wang

**Affiliations:** Department of Obstetrics and Gynecology, The First Affiliated Hospital of Guangzhou Medical University, Guangzhou, China

**Keywords:** endometriosis, endometrium, infiltrating immune cells, infertile, inflammatory

## Abstract

Endometriosis is an oestrogen-dependent chronic inflammatory process with primary symptoms including dysmenorrhea, chronic pelvic pain, and infertility. The immune environment of the endometrium is essential for successful embryo implantation and ongoing pregnancy. In this study, we assessed the composition, density, and distribution of infiltrating immune cells in the endometria of women with endometriosis. Gene expression profiles of endometrial samples were downloaded from the Gene Expression Omnibus (GEO) database. We found that the TNF signalling pathway, the IL-17 signalling pathway, and the MAPK signalling pathway were significantly enriched in the eutopic endometria of women with endometriosis. The fractions and proportion of infiltrating immune cells were estimated by the CIBERSORT, MCP-counter, and ImmuCellAI methods. We found that the proportions of CD8^+^ T cells, activated NK cells, and follicular helper T cells were significantly higher in the endometria of women with endometriosis than in the endometria of normal controls, while the proportions of M2 macrophages and resting mast cells were significantly lower in the eutopic endometria. In GSE120103 (n = 36), we found that elevated CD8^+^ T cells in endometriosis increased the risk of infertility (P = 0.0019). The area under the receiver operating characteristic (ROC) curve (AUC) of CD8^+^ T cells to distinguish fertile and infertile endometriosis was 0.914. In clinical samples (n = 40), we found that the proportions of CD8^+^ T cells and CD56^+^ NK cells were significantly higher in the eutopic endometria of women with endometriosis than in the endometria of normal controls, while the proportion of CD163^+^ macrophages were lower in the eutopic endometria. The AUCs of CD8^+^ T cells and CD163^+^ macrophages were 0.727 and 0.833, respectively, which indicated that CD8 and CD163 were potential diagnostic markers for endometriosis. In conclusion, our results demonstrated that increased CD8^+^ T cells and CD56^+^ NK cells and decreased CD163^+^ macrophages within the eutopic endometria of women with endometriosis reveal a proinflammatory feature in the endometrial immune environment and that elevated CD8^+^ T cells increase the risk of infertility in women with the disease.

## Introduction

Endometriosis, a condition in which endometrial-like tissue aberrantly grows outside the uterus, is characterized by an oestrogen-dependent chronic inflammatory process and symptoms including dysmenorrhea, chronic pelvic pain, and infertility (1). Endometriosis affects nearly 10–15% of women of reproductive age (2), and 30–50% of women with endometriosis have infertility (3). Therefore, it is important to investigate the mechanism of endometriosis-associated infertility. Endometriosis affects the quality of embryos, the function of fallopian tubes and embryo transportation, and the endometrial receptivity and embryo implantation (4); these abnormalities likely all impact fertility, which leads to infertility or pregnancy loss. The immune environment of the endometrium is a key factor for endometrial receptivity and is essential for successful embryo implantation and ongoing pregnancy. The eutopic endometria of women with endometriosis manifest progesterone resistance and oestrogen dominance, resulting in disturbed decidualization and aberrant inflammation (5, 6).

Previous studies have indicated that the immune environment within the eutopic endometria of women with endometriosis is different from that within normal endometria, such as the components of immune cells (7–9) and the activation of immune-inflammation signalling pathways (10, 11). However, the proportion and distribution of different subtypes of immune cells within the eutopic endometria of women with endometriosis remain controversial. For example, four groups have reported a greater abundance of macrophages (Møs) in the endometria of women with endometriosis in the proliferative phase (12) or independent of the hormonal milieu (7, 9, 13) compared with normal controls, a finding that was not confirmed by another group (14). Most previous studies have adopted an immunohistochemistry or flow cytometry method to evaluate the landscape of infiltrating immune cell subtypes. Because of the limited number of biomarkers in one experiment, to date, there are no data providing a complete phenotyping of immune cells within the eutopic endometria of women with endometriosis. Recently, a transcriptome meta-analysis by the xCell algorithm revealed the differences in the immune profile between eutopic endometria and stage I–II and III–IV endometriosis independent of the hormonal milieu (9). However, the study lacks validation in clinical specimens.

With the boom in high-throughput technologies over the last decade, including microarrays and RNA sequencing, large‐scale transcriptome data was produced, which provides opportunities for estimating the abundance of immune cells using gene expression profiles. To date, several methods, including CIBERSORT, xCell, EPIC, TIMER, MCP-counter, and ImmuCellAI, have been developed for estimating immune cells from bulk transcriptome data of different diseases, including cancer and benign disease (15–17). CIBERSORT is a bioinformatics tool that uses the deconvolution method to characterize cell subsets of interest in high-dimensional genomic data derived from bulk tissue samples (4, 15). The Microenvironment Cell Populations-counter (MCP-counter) method and Immune Cell Abundance Identifier (ImmuCellAI) are both gene set signature‐based methods for estimating the abundance of tissue-infiltrating immune cell populations from transcriptomic data (18, 19). Compared with CIBERSORT and the MCP-counter method, ImmuCellAI can be used to estimate the abundance of 18 T‐cell subsets, such as iTreg, Tc, and exhausted T cells (Tex) (19). Gene Expression Omnibus (GEO) provides a large amount of transcriptome data of different diseases, including eutopic endometria and normal controls.

In this study, we assessed the composition, density, and distribution of infiltrating immune cells in the endometria of women with endometriosis by CIBERSORT, MCP-counter, and ImmuCellAI methods. Considering the features of infiltrating immune cells throughout the menstrual cycle, all samples enrolled in this study were in proliferation and early secretory phases so that the data between the two groups were comparable. Then, GSE120103 was used to analyse the relationship between infiltrating immune cells and infertility in women with endometriosis. Next, the results of transcriptomic analysis were validated by immunochemistry and immunofluorescence in clinical paraffin-embedded endometrial specimens. We believe that immune infiltration analysis based on transcriptome data by multiple methods is inexpensive and easily available and is of greater reference value because it is based on clinical specimen validation.

## Materials and Methods

### Data Collection and Processing

Gene expression profiles of endometria with or without endometriosis were downloaded from the GEO database (https://www.ncbi.nlm.nih.gov/geo/). Eligibility criteria included samples of eutopic endometria from women with endometriosis and from healthy controls without any pathological condition, samples within the proliferation and early secretory phases of the menstrual cycle, and samples containing both glandular and stromal components. GSE25628, GSE6364, and GSE51981, containing 67 samples of eutopic endometria and 40 normal controls, were included in this study (**Table 1**). The datasets were combined and processed by the sva package to remove batch effects because the datasets came from multiple cohorts and array platforms (20) and then were normalized by the limma package before further analysis (21). GSE120103 contains 36 endometrial samples of fertile or infertile women with or without endometriosis (22), which were downloaded and processed by the GEOquery package. The raw data are available in NCBI-GEO, from which they can be downloaded and analysed.

**Table 1:** Gene expression datasets used in this study.

GEO accession	Platform	Experiment type	EM (n)	Normal (n)	Tissue	Year
GSE6364	GPL570	mRNA array	12	8	Endometrium	2006
GSE25628	GPL571	mRNA array	8	6	Endometrium	2010
GSE51981	GPL570	mRNA array	47	26	Endometrium	2013
GSE120103	GPL6480	mRNA array	18	18	Endometrium	2018
GSE130435	GPL20301	mRNA sequencing	4	6	Macrophages M2	2019

### Estimation of Infiltrating Immune Cells

The infiltrating immune cells in the endometrium were estimated by the CIBERSORT, MCP-counter, and ImmuCellAI algorithms. CIBERSORT method was performed by its package within R as previously described (15). The number of permutations of the default signature matrix was set to 1,000. Only samples with a CIBERSORT P<0.05 were deemed qualified for further analysis. Only types of infiltrating immune cells that could be detected in most of the samples (a 75% cut-off) were included for further analysis. The MCP-counter was performed within R as previously described (18), and a normalized gene matrix was loaded and further analysed. The normalized gene matrix was also analysed by ImmuCellAI online (http://bioinfo.life.hust.edu.cn/ImmuCellAI#!/) (19), immune cell abundance in all samples was estimated, and the results were visualized by pheatmap and ggplot2 packages within R. The infiltrating immune cells in the infertile cohort (GSE120103) were estimated by MCP-counter method because its normalized matrix contains negative values.

### Clinical Samples

Between January 2018 and December 2019, 40 archival formalin-fixed paraffin-embedded endometrial specimens were obtained from the First Affiliated Hospital of Guangzhou Medical University. This study was approved by the Institutional Research Ethics Committee and is compliant with the principles set forth by the Declaration of Helsinki principles. Informed consent was obtained from each patient before using the samples. The specimens included 20 cases of eutopic endometria with endometriosis and 20 normal controls. All enrolled patients were in the proliferation and early secretory phases of the menstrual cycle, which were confirmed by the patient’s menstrual cycle and pathological examination. Two experienced pathologists who had no prior knowledge of the patient data reviewed the slides for all cases to confirm the previous diagnosis independently. Serial sections were prepared for haematoxylin and eosin (HE) staining, immunohistochemistry (IHC) staining, and immunofluorescence staining.

### Immunohistochemistry Staining and Analysis

IHC was performed as previously described (23, 24). Briefly, the paraffin-embedded samples were sectioned at 4 μm thickness. Antigen retrieval was performed using target retrieval solution Tris-EDTA buffer pH 9.0 (for CD56 and CD117) and pH 8.0 (for CD45) or citrate buffer pH 6.0 (for CD8 and CD163) with a pressure cooker for 3–5 min. Endogenous peroxidase activity was blocked by incubating for 30 min in 3% H2O2 at room temperature. Non-specific binding was blocked with 5% BSA for 30 min at room temperature. The tissues were incubated with antibodies against CD45 (1:200, ZM-0183, Zsgb-Bio), CD8 (1:200, ab237709, Abcam), CD56 (1:100, ZM-0057, Zsgb-Bio), CD117 (1:200, ab32363, Abcam), and CD163 (1:200, ab156769, Abcam) overnight at 4°C, and immune detection was performed using DAB kits (PV-9001 and PV-9002, Zsgb-Bio) according to the manufacturer’s instructions. Each specimen was first screened at low magnification (×100), and the five fields with the greatest number of positively stained cells were selected for further analysis. The mean cell counts in these five fields for each case were estimated at high-power (×200) magnification and determined by two independent pathologists who were blinded to the patients’ pathological and clinical status. To confirm the reproducibility, 25% of the slides were chosen randomly and scored twice. All duplicates were evaluated in a similar manner. CD45 (lymphocyte common antigen, LCA) is a receptor-linked protein tyrosine phosphatase that is expressed on all leukocytes (25). Therefore, the counts of CD45 in every sample were used to calculate the fraction and proportion of immune cell subsets. The mean ± SD are shown.

### Immunofluorescence Staining

Clinical paraffin-embedded samples were used for immunofluorescence staining. Briefly, 4-μm-thick paraffin sections were deparaffinized and rehydrated. Antigen retrieval was performed using target retrieval solution Tris-EDTA buffer (pH 9.0) and citrate buffer (pH 6.0) with a pressure cooker for 10–15 min. The non-specific binding was blocked with 5% BSA for 30 min at room temperature. Subsequently, the sections were simultaneously incubated with rabbit monoclonal anti-CD8 antibodies (1:200, ab237709, Abcam) and mouse monoclonal anti-CD163 (1:200, ab156769, Abcam) in a humidified chamber overnight at 4°C. Alexa Fluor^®^ 647 conjugated donkey anti-rabbit antibodies (1:200, ab150075, Abcam) and CY3 conjugated donkey anti-mouse antibodies (1:200, GB21401, Servicebio) were used as secondary antibodies. DAPI was then used to counterstain the nuclei, and images were obtained by fluorescence microscopy (Leica DM6).

### Differentially Expressed Gene Analysis and Pathway Enrichment Analysis

Differentially expressed genes (DEGs) between the eutopic endometria and normal controls were identified by the limma package in R. DEGs were identified based on a 5% adjusted p value and a 1.261 log fold change (logFC) cut-off. LogFC cut-off was defined as the mean of the sums of the absolute value of logFC and its two standard deviations in the whole expression matrix. Volcano plots and heatmaps are used to display the meaningful DEGs. KEGG pathway and GO term enrichment analyses for DEGs are displayed by the clusterProfiler package as previously published (26).

### Statistical Analysis

All data were statistically analysed using SPSS 24.0 software (IBM Corporation, Armonk, NY, USA) and are expressed as the mean ± standard deviation. Student’s t-test and the Wilcoxon rank-sum test were used to evaluate differences between two groups. Differences were considered significant at bilateral P values < 0.05.

## Results

### Gene Expression Signatures of the Eutopic Endometria of Women With Endometriosis

**Figure 1:**
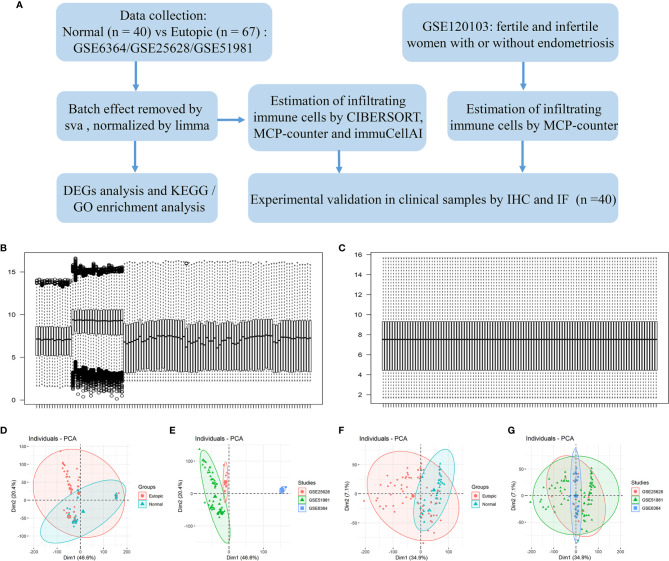
The study flowchart and gene expression after combining data from the datasets and identifying and removing the batch effect. **(A)** The study flowchart. **(B, C)** Boxplots show the intensity of the log2-transformed gene expression before **(B)** and after **(C)** batch effect removal. **(D–G)** Scatter plots show the principal component analysis (PCA) analysis of normalized gene expression data before **(D, E)** and after **(F, G)** batch effect removal by Combat. Ellipses underlying assumptions about the distribution of the data were drawn considering a multivariate t-distribution and a confidence level of 0.95. GO, Gene Ontology; KEGG, Kyoto Encyclopedia of Genes and Genomes; DEGs, differentially expressed genes. IHC, immunohistochemistry; IF, immunofluorescence.

A flowchart of the analysis procedures, including transcriptomic analysis and experimental validation, for this study is shown in **Figure 1A**. A total of 107 endometrial samples (67 eutopic endometria and 40 normal controls) and 12,402 filtered and normalized genes were included in this study (**Table 1**). The batch effects of the three GEO series (GSE25628, GSE6364, and GSE51981) were processed by the Combat function in the sva package and normalized by limma packages. Boxplots and PCA scatter plots show the normalized gene expression data before and after batch effect removal (**Figures 1B–G**). Ninety-eight upregulated and 218 downregulated genes were identified in the eutopic endometria of women with endometriosis compared with the endometria of normal controls (**Supplemental Figure 1A**). According to KEGG pathway and Gene Ontology (GO) term enrichment analyses of the DEGs, the TNF signalling pathway, IL-17 signalling pathway, and MAPK signalling pathway were significantly enriched (**Supplemental Figures 1C–F**), which indicated that the eutopic endometria of women with endometriosis have prominent inflammatory features.

### The Signatures of Infiltrating Immune Cells in the Eutopic Endometria of Women With Endometriosis

**Figure 2:**
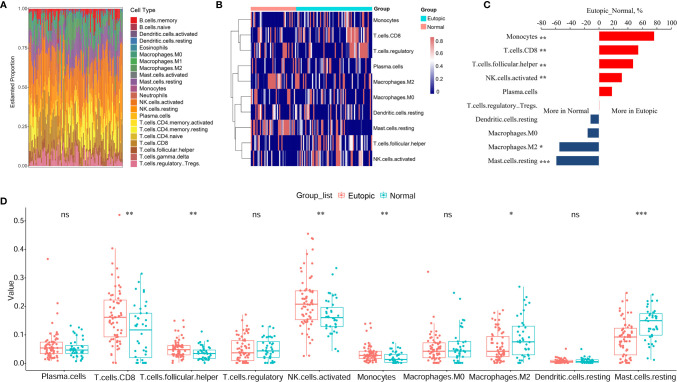
The estimation of infiltrating immune cells in the endometria with or without endometriosis in the GEO cohort by CIBERSORT. **(A)** The stacked bar plot figure shows the inferred composition of 22 immune cell subsets in the endometria. **(B)** The heatmap shows the estimated proportions of 10 major immune cell subsets (plasma cells, CD8 T cells, follicular helper T cells, regulatory T cells, activated NK cells, monocytes, M0 macrophages, M2 macrophages, resting dendritic cells, and resting mast cells) in the endometria. **(C)** Comparisons of immune cells more in endometriosis or more in normal samples. **(D)** Boxplots comparing the proportions of 10 major immune cell subsets between normal and endometriosis samples. Data were assessed by the Wilcoxon rank-sum test. *P < 0.05, **P < 0.01, and ***P < 0.001. ns, no significance.

First, we estimated the composition of infiltrating immune cells in the endometria using the normalized gene matrix of 107 samples by the CIBERSORT algorithm. The 22 types of infiltrating immune cells inferred by CIBERSORT include B cells, T cells, natural killer cells, macrophages, dendritic cells, eosinophils, and neutrophils and are shown in a stacked bar plot in **Figure 2A**. After filtering by the CIBERSORT p value and the cut-off, we obtained 10 major immune cell subsets (plasma cells, CD8^+^ T cells, follicular T helper cells, regulatory T cells, activated NK cells, monocytes, M0 macrophages, M2 macrophages, resting dendritic cells, and resting mast cells) in the endometria (**Figure 2B**). Six of 10 types of immune cells were significantly changed between the eutopic endometria of women with endometriosis and the endometria of normal controls (**Table 2**). Compared to the results for the normal controls, the proportions of CD8^+^ T cells, activated NK cells, follicular helper T cells, and monocytes were significantly higher in the eutopic endometria of women with endometriosis (P = 0.0033; P = 0.0029; P = 0.0086; and P = 0.0054), while the proportions of M2 macrophages and resting mast cells were significantly lower in the eutopic endometria of women with endometriosis (P = 0.0336; P = 5.9e-05; **Figures 2C, D**).

**Table 2:** The proportions of 10 major infiltrating immune cell subsets in the endometrium with or without endometriosis estimated by CIBERSORT.

Types of immune cells	Eutopic (Mean ± SD)	Normal (Mean ± SD)	P values
Plasma.cells	0.0607 ± 0.0524	0.0520 ± 0.0329	0.4228
T.cells.CD8	0.1659 ± 0.0968	0.1086 ± 0.0939	0.0033
T.cells.follicular.helper	0.0502 ± 0.0311	0.0349 ± 0.0232	0.0086
T.cells.regulatory (Tregs)	0.0476 ± 0.4356	0.0484 ± 0.0384	0.0720
NK.cells.activated	0.2130 ± 0.0915	0.1650 ± 0.0609	0.0029
Monocytes	0.0333 ± 0.0296	0.0193 ± 0.0191	0.0054
Macrophages.M0	0.0504 ± 0.0490	0.0597 ± 0.0599	0.6965
Macrophages.M2	0.0591 ± 0.0508	0.0896 ± 0.0717	0.0336
Dendritic.cells.resting	0.0066 ± 0.0077	0.0073 ± 0.0091	0.9303
Mast.cells.resting	0.0847 ± 0.0619	0.1360 ± 0.0556	5.9e-05

**Figure 3:**
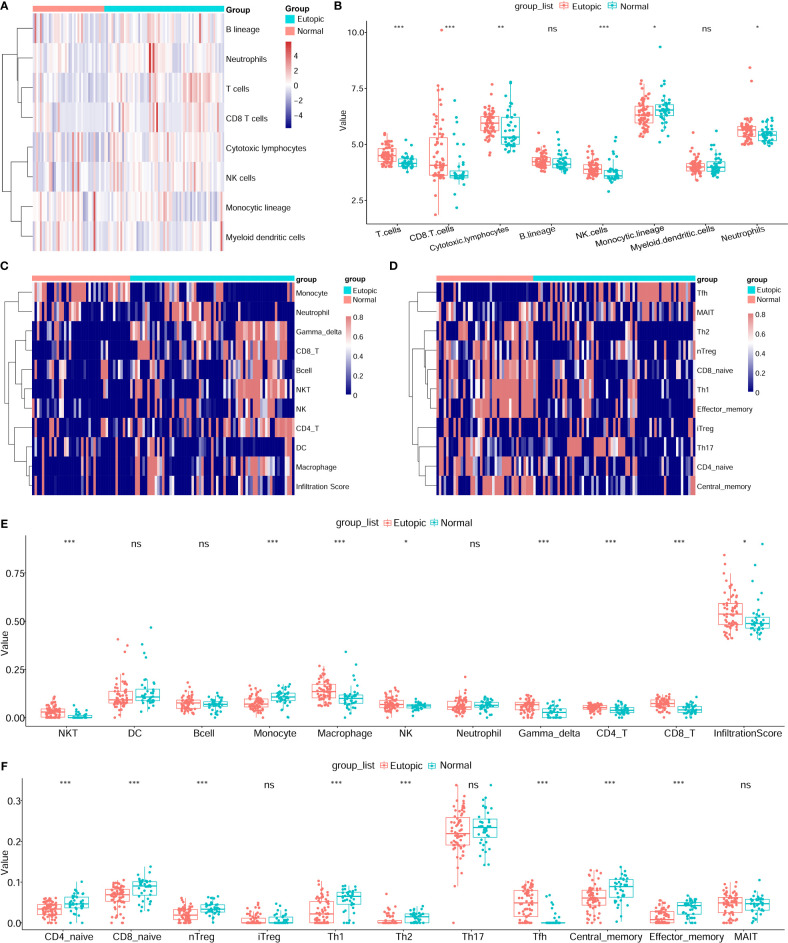
Estimation of infiltrating immune cells by the MCP-counter and immuCellAI methods. **(A, B)** The results of the MCP-counter method: the heatmap shows the absolute abundances of eight immune cell subsets **(A)** and boxplots comparing the abundances of eight immune cell subsets between normal and endometriosis samples. **(C–F)** The immuCellAI results: the heatmap shows the proportions of 10 major types of immune cells and the infiltration scores **(C)** and boxplots comparing the proportions of 10 immune cell subsets between normal and endometriosis samples. The subsets of CD4+ T cells and CD8+ T cells are also shown by the heatmap **(D)** and boxplots **(F)**. Data were assessed by the Wilcoxon rank-sum test. *P < 0.05, **P < 0.01, and ***P < 0.001. ns, no significance.

Then, the infiltrating immune cells within the 107 samples were also estimated by MCP-counter and ImmuCellAI methods. For the MCP-counter method, we found that T cells (P = 1.6e-6), CD8^+^ T cells (P = 0.00018), cytotoxic lymphocytes (P = 0.0038), and NK cells (P = 0.00016) were elevated in the eutopic endometria of patients with endometriosis compared to the endometria of healthy controls (**Figures 3A, B**), while monocytic lineage was decreased in the eutopic endometrium samples (P = 0.04699). For ImmuCellAI, the infiltration score of the eutopic endometria was higher than that of the controls (P = 0.01058). In the eutopic endometrium, NK cells (P = 0.039), CD4^+^ T cells (P = 0.001), and CD8^+^ T cells (P = 7.2e-8) were also higher than those in healthy controls (**Figures 3C, E**). Moreover, the subsets of CD4^+^ T cells and CD8^+^ T cells were further compared between the two groups, and we found that follicular helper T cells (Tfhs) were significantly higher in the eutopic endometrium samples compared to those of healthy controls (P = 1.39e-9), while naive CD4^+^ T cells (P = 0.00013), naive CD8^+^ T cells (P = 7.6e-5), nTreg (P = 4.6e-5), Th1 (P = 6.9e-6) and Th2 (P = 0.00014), central memory (P = 0.00076), and effector memory T cells (P = 1.1e-6) were lower in the eutopic endometrium samples compared to those of healthy controls (**Figures 3D, F**).

### The Relationship Between Infiltrating Immune Cells and Infertility in Patients With Endometriosis

**Figure 4:**
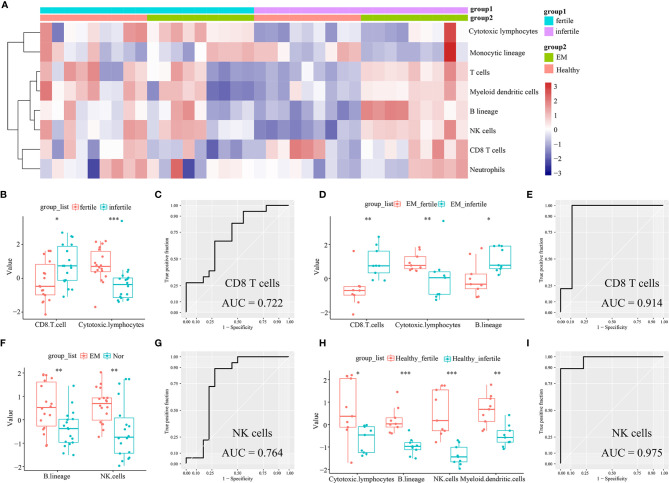
Estimation of infiltrating immune cells in the endometria of fertile and infertile cohorts (GSE120103) by the MCP-counter method. **(A)** The heatmap shows the absolute abundance of eight immune cell subsets. **(B)** Boxplots show differential infiltrating immune cells (CD8 T cells and cytotoxic lymphocytes) between fertile (n = 18) and infertile (n = 18) groups. **(C)** The ROC curve of CD8+ T cells in fertile and infertile cohorts (n = 36). **(D)** Boxplots show differential infiltrating immune cells (CD8 T cells, cytotoxic lymphocytes, and B lineage) between fertile (n = 9) and infertile (n = 9) groups in patients with endometriosis. **(E)** The ROC curve of CD8+ T cells in patients with endometriosis (n = 18). **(F)** Boxplots showing differentially infiltrating immune cells (NK cells and B lineage) in women with (n = 18) or without (n = 18) endometriosis. **(G)** The ROC curve of NK cells in women with or without endometriosis (n = 36). **(H)** Boxplots show differential immune cells (cytotoxic lymphocytes, B lineage, NK cells, and myeloid dendritic cells) between fertile (n = 9) and infertile (n = 9) groups in healthy women. **(I)** The ROC curve of NK cells in a cohort of healthy women (n = 18). Data were assessed by the Wilcoxon rank-sum test. *P < 0.05, **P < 0.01, and ***P < 0.001. AUC, the area under the ROC curve.

The infiltrating immune cells in the 36 endometrial tissue samples of fertile or infertile women with or without endometriosis (GSE120103) were estimated by the MCP-counter method. We found that CD8^+^ T cells were higher in infertile women (n = 18) than in fertile women (n = 18) (P = 0.0224), while cytotoxic lymphocytes were lower in the infertile group (P = 0.0005, **Figures 4A, B**). The area under the ROC curve (AUC) of CD8^+^ T cells to distinguish fertile and infertile women was 0.722 (**Figure 4C**). In patients with endometriosis, CD8^+^ T cells were also higher in infertile women (n = 9) than in fertile women (n = 9) (P = 0.0019), and the AUC of CD8^+^ T cells was 0.914 (**Figure 4E**). In both the endometriosis and healthy cohorts, cytotoxic lymphocytes were lower in infertile patients (P = 0.0078; P = 0.02443, **Figures 4D, H**). B lineage cells were higher in the eutopic endometria of patients with endometriosis (n = 18) than in the endometria of healthy controls (n = 18) (P = 0.0071, **Figure 4F**). In patients with endometriosis, B lineage cells were higher in the infertile group than in the fertile group (P = 0.0106, **Figure 4D**). NK cells were higher in the group with eutopic endometria than in the healthy controls (P = 0.0064, **Figure 4F**). The AUC of NK cells to distinguish endometriosis and healthy women was 0.764 (**Figure 4G**). In healthy women, NK cells were higher in fertile women than in infertile women (P = 0.00016, **Figure 4H**). The AUC of NK cells to distinguish between fertile and infertile women in healthy women was 0.975 (**Figure 4I**).

### Validation of the Density and Distribution of Endometrial Immune Cells in Clinical Samples

**Figure 5:**
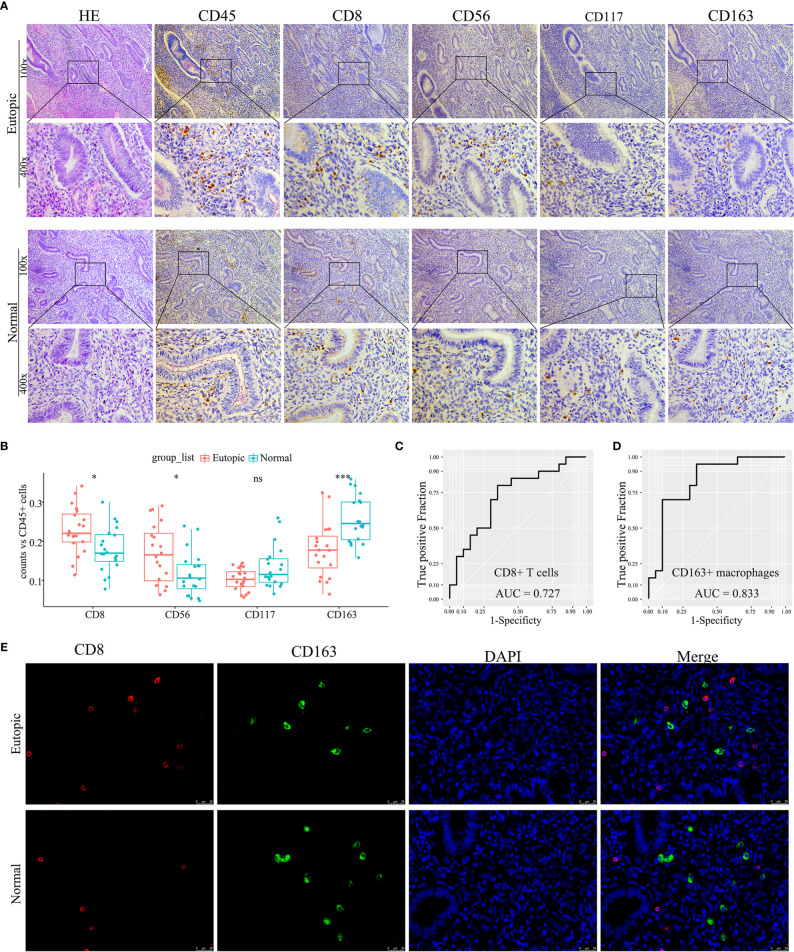
Experimental validation of infiltrating immune cells in clinical endometrial samples. **(A)** Representative photographs of haematoxylin-eosin (HE) staining and immunohistochemistry (CD45, CD8, CD56, CD117, and CD163). Magnifications at 100× and 400× are shown. **(B)** Boxplots comparing the proportions of four immune cell subsets between normal and endometriosis tissues. Data were assessed by the Wilcoxon rank-sum test, and P values are labelled. ns, no significance. **(C, D)** The ROC curve of CD8^+^ T cells and CD163^+^ macrophages in clinical samples. AUC, the area under the ROC curve. **(E)** Representative photographs of immunofluorescence (CD8, CD163, and DAPI). Magnification was 400×. *P < 0.05, ***P < 0.001, ns, no significance.

To confirm further the density and distribution of immune cells in the endometrium, the differentially infiltrating immune cells were validated by immune cell biomarkers in clinical paraffin-embedded endometrial specimens (n = 40) by immunohistochemistry using serial sections. CD8 is for CD8^+^ T cells, CD56 is for NK cells, CD117 is for mast cells, and CD163 is for M2 macrophages. CD45 was also stained and used to calculate the proportion of each immune cell subset. We found that the proportions of CD8^+^ T cells and CD56^+^ NK cells were significantly higher in the eutopic endometria of women with endometriosis than in women with normal endometria (0.2292 ± 0.0591 *vs* 0.1790 ± 0.0562, P = 0.0132; 0.1686 ± 0.0745 *vs* 0.1163 ± 0.056, P = 0.0227; **Figures 5A, B** and **Table 3**), while CD163^+^ macrophages were lower in the eutopic endometria of women with endometriosis compared to their counterparts (0.1774 ± 0.0685 *vs* 0.2555 ± 0.0588, P = 0.0003; **Figures 5A, B** and **Table 3**). These results verified in clinical samples were consistent with those estimated by the CIBERSORT, MCP-counter, and ImmuCellAI algorithms. However, CD117^+^ mast cells were not significantly different between the groups in the clinical samples (**Figures 5A, B**). The areas under the ROC curves (AUCs) of CD8^+^ T cells and CD163^+^ macrophages were 0.727 and 0.833, respectively, which indicated that CD8 and CD163 were potential diagnostic markers for endometriosis (**Figures 5C, D**). Then, CD8 and CD163 were simultaneously stained by immunofluorescence in the endometrium samples, which again confirmed the distribution of CD8^+^ T cells and CD163^+^ macrophages in the endometria, as shown in **Figure 5E**.

**Table 3:** The proportions of infiltrating immune cells in the endometrium between normal and endometriosis.

Types of immune cells	Eutopic (Mean ± SD)	Normal (Mean ± SD)	P values
CD8^+^ T cells	0.2292 ± 0.0591	0.1790 ± 0.0562	0.0132
CD56^+^ NK cells	0.1686 ± 0.0745	0.1163 ± .0560	0.0227
CD117^+^ Mast cells	0.1026 ± 0. 0279	0.1338 ± 0.0538	0.0718
CD163^+^ Macrophages	0.1774 ± 0.0685	0.2555 ± 0.0588	0.0003

## Discussion

Although a number of studies and great efforts have been made in the past few decades, to date, the pathogenesis of endometriosis is poorly understood. Genetics and environmental factors are key drivers, and the immune system is believed to play a major role in its pathophysiology and symptomatology (2, 27). The inflammatory features of endometriosis manifest not only in the pelvic cavity and ectopic lesions but also in the eutopic endometrium (28). Previous studies have reported that changes in endometrial immune status directly or indirectly contribute to disease establishment and the inhospitable environment for embryo implantation, which can lead to an increased risk of infertility and adverse pregnancy outcomes in women with endometriosis (8). Here, in combination with bioinformatics analysis and clinical sample validation, we provided a clear understanding of the composition, density, and distributions of infiltrating immune cells within the eutopic endometria of patients with endometriosis. We found that CD8^+^ T cells and CD56^+^ NK cells were higher in the eutopic endometria of women with endometriosis than in the endometria of normal controls, while CD163^+^ macrophages were lower in the eutopic endometria. Elevated CD8^+^ T cells and NK cells increase the risk of infertility. According to the results of differentially expressed gene analysis and KEGG pathway and GO term enrichment analyses, the TNF signalling pathway, IL-17 signalling pathway, and MAPK signalling pathway were significantly enriched in the eutopic endometria of women with endometriosis, which indicated a proinflammatory immune status in the eutopic endometria of women with endometriosis.

In general, T cells are effector cells of the adaptive immune system (29). According to the results of the CIBERSORT, MCP-counter, and ImmuCellAI methods, we found that the abundances of T cells, CD4^+^ T cells, follicular helper T (Tfh) cells, and CD8^+^ T cells in the eutopic endometria of women with endometriosis were higher than those in the endometria of normal controls. Tfh cells are a specialized subset of CD4^+^ T cells that play an important role in the adaptive immune response (30). There are few reports about the functions of Tfh cells in endometriosis. Luan et al. reported that memory Tfh cells with the CD4^+^ CXCR5^+^ PD1^+^ CCR7^−^ and CD4^+^ CXCR5^+^ PD-1^+^ ICOS^+^ phenotypes showed a significant increase in recurrent spontaneous abortion (RSA) patients compared to women with a normal pregnancy who had chosen termination (31). Whether elevated Tfh cells in the eutopic endometria of women with endometriosis increase the risk of infertility warrants further research. The main function of CD8^+^ T cells is to monitor all the cells of the body, ready to destroy those that may threaten the homeostasis of the host (32). A previous study has reported that CD8^+^ T cells in ectopic lesions are higher in number than those in the eutopic endometria of women with endometriosis (33) and do not vary with the hormonal milieu (34). However, whether the numbers of endometrial CD8^+^ T cells differ between women with and without endometriosis remains to be determined. Herein, we found that CD8^+^ T cells were higher in the eutopic endometria of patients with endometriosis than in the endometria of normal controls and that elevated CD8^+^ T cells increased the risk of infertility in patients with endometriosis. In clinical samples, we validated the density and distribution of elevated CD8^+^ T cells in the eutopic endometria of women with endometriosis. The AUC of CD8^+^ T cells was 0.727, which indicated that CD8 is a potential diagnostic marker to distinguish endometriosis and normal controls.

Previous studies reported that uterine natural killer (uNK) cells are the predominant leukocyte population in the human endometrium (35), comprising 30–40% of total leukocytes in the proliferative phase and up to 70% in the secretory phase (36, 37). We found that the total proportion of NK cells (resting and activated subtypes) in 22 immune cell types was approximately 29.0%. As most of the samples enrolled were in the proliferation phase, the content of NK cells was consistent with that reported in the literature. The proportion of activated NK cells was significantly higher in the eutopic endometria of women with endometriosis than in the endometria of normal controls, while resting NK cells were not significantly different between the groups. In GSE120103, we also found that the abundance of NK cells in the eutopic endometria of women with endometriosis was higher than in the endometria of healthy controls. NK cells act as the first line of defence against viral infections and tumour growth and are important for normal tissue homeostasis (38). Previous studies have reported that uNK cells are CD9^+^CD3^−^CD56^High^CD16^Low^, whereas peripheral blood NK cells are CD9^+^CD3^−^CD56^Low^CD16^High^ (39, 40). Therefore, we selected anti-CD56 antibodies to detect NK cells in clinical endometrium samples, and we confirmed that CD56^+^ NK cells were significantly elevated in the endometria of patients with endometriosis compared with those of normal controls. Moreover, uNK cells are key players in embryo implantation because they produce and secrete angiogenic factors, including vascular VEGF and angiotensin 2 (ANG2) that promote the maturation of endometrial blood vessels, which is important for successful implantation and pregnancy establishment (41, 42). It is likely that defects in NK cell activity that contribute to the pathogenesis of endometriosis will significantly impact fertility. In healthy women, we found that NK cells were higher in fertile women than in infertile women, and the AUC of the ROC curve was 0.975. However, this finding was not significant in women with endometriosis, which may be due to the limited sample size of GSE120103. A previous study reported that more immature uNK cells (stage I–II) were found in the eutopic endometria of women with endometriosis than in the endometria of normal controls, which is associated with infertility or recurrent pregnancy loss (43). CD56^High^ NK cells were first considered “immunoregulatory” by Cooper et al. because of the increased production of cytokines and reduced cytotoxicity compared to CD56^Low^ NK cells (44). Although the absolute numbers of uNK cells are elevated in endometriosis eutopic endometrium, they are likely dysfunctional, and their cytotoxicity is diminished, consistent with their CD56^High^CD16^Low^ phenotype and lack of activating NKp30 and NKp44 receptors (45).

Macrophages have been widely studied in endometriosis ectopic lesions, but little is known about them in the eutopic endometrium and how they (and other immune cells) contribute to disease establishment and the inhospitable environment for embryo implantation, which leads to an increased risk of infertility and adverse pregnancy outcomes in women with disease (8). Macrophages are classified as “classically activated” M1 macrophages or “alternatively activated” M2 macrophages depending on the activation state and surface markers (46). We found that the number of M2 macrophages was lower in the endometria of patients with endometriosis than in normal controls. A previous study found that M1 macrophages were higher in stage I–II endometriosis than in healthy controls, whereas M2 macrophages were elevated in the eutopic endometria of women with stage III–IV endometriosis (9). However, we found that the abundance of M1 or M2 macrophages between stage I–II and stage III–IV endometriosis was not significantly different. In GSE130435, we found that the differentially expressed genes of M2 macrophages between endometriosis and normal controls were mainly enriched in immune system processes and defence responses to other organisms (**Supplemental Figure 3**). A previous study has also reported that M2 macrophages are the predominant phenotype in healthy endometria (47), while the endometrial macrophage M2 population in women with endometriosis was lower in all cycle phases than in controls (7). In addition, M1 macrophages have proinflammatory properties, and M2 macrophages have anti-inflammatory properties. Therefore, more M2 macrophages in the endometria of healthy women maintain an anti-inflammatory environment, which allows embryo implantation. In contrast, levels of IL-1, IL-6, and IL-8 and hepatocyte growth factor (HGF) are increased in the endometria of women with endometriosis compared with the endometria of normal controls (48), and elevated levels of monocyte/macrophage-activating chemoattractant protein-1 (MCP-1) (49) and macrophage inhibitory factor (50) further promote a proinflammatory environment in the endometria of women with disease.

Overall, it is a practicable strategy to profile the signatures of infiltrating immune cells in the endometria between women with or without endometriosis by integrating the transcriptome matrix, using the CIBERSORT, MCP-counter, and ImmuCellAI methods and validating them in clinical samples. Our data revealed an altered proinflammatory immune environment in the endometria of women with endometriosis compared to those without endometriosis, along with other abnormalities in non-immune cells in this tissue, which are believed to contribute to endometriosis establishment, pathophysiology, and known adverse reproductive outcomes. We first demonstrated the abundance of CD8^+^ T cells in the eutopic endometria of women with endometriosis, and we found a relationship between CD8^+^ T cells and infertility in women with endometriosis. This study helps us to understand the mechanism of infertility in endometriosis and provides potential applications for the diagnosis and treatment of this disease. The results of this study highlight the important roles of the endometrial immune environment in endometriosis and potential therapeutic targets in treating this disease, providing opportunities for future research.

In conclusion, we profiled the signatures of infiltrating immune cells in the eutopic endometria of women with endometriosis. Our results demonstrated that increased CD8^+^ T cells and CD56^+^ NK cells and decreased CD163^+^ macrophages within the eutopic endometria of women with endometriosis reveal a proinflammatory feature in the endometrial immune environment and that elevated CD8^+^ T cells increase the risk of infertility in women with the disease.

## Data Availability Statement

The datasets presented in this study can be found in online repositories. The names of the repository/repositories and accession number(s) can be found below: https://www.ncbi.nlm.nih.gov/, GSE6364

https://www.ncbi.nlm.nih.gov/, GSE25628

https://www.ncbi.nlm.nih.gov/, GSE51981

https://www.ncbi.nlm.nih.gov/, GSE120103

https://www.ncbi.nlm.nih.gov/, GSE130435

## Ethics Statement

The studies involving human participants were reviewed and approved by the Medical Ethics Committee of the First Affiliated Hospital of Guangzhou Medical University. The patients/participants provided their written informed consent to participate in this study.

## Author Contributions

X-GW, J-JC, H-LZ, YW, FL, JS, H-ZW, H-QX, and WW conceived and designed the project. X-GW, J-JC, and H-LZ studied the concept and detailed design, draft preparation, data collection, analysis, interpretation, and critical revision of the manuscript for important intellectual content. YW, FL, JS, and H-ZW helped to prepare figures and/or tables. H-QX and WW supervised the project and contributed to the writing and revision of the manuscript. All authors contributed to the article and approved the submitted version.

## Conflict of Interest

The authors declare that the research was conducted in the absence of any commercial or financial relationships that could be construed as a potential conflict of interest.

## Publisher’s Note

All claims expressed in this article are solely those of the authors and do not necessarily represent those of their affiliated organizations, or those of the publisher, the editors and the reviewers. Any product that may be evaluated in this article, or claim that may be made by its manufacturer, is not guaranteed or endorsed by the publisher.

## Supplementary Material

Supplementary Figure 1 Differential expression gene analysis of the endometrium between normal and endometriosis tissues and KEGG pathway and GO term enrichment analyses. **(A)** Volcano plot analysis of differentially expressed genes (DEGs) in the endometrium between normal and endometriosis tissues. **(B)** The heatmap of Top100 DEGs. **(C)** Bar plots of KEGG pathway enrichment analysis. **(D–F)** Dotplots of GO term enrichment analyses, including biological process **(D)**, cellular component **(E)**, and molecular function **(F)**.

Supplementary Figure 2 Estimation of infiltrating immune cells in the endometria of ASRM clinical stages of endometriosis (GSE51981) by CIBERSORT. **(A)** The heatmap showing the estimated proportions of 12 major immune cell subsets in the endometria. **(B)** Boxplots comparing the proportions of 12 major immune cell subsets between minimal/mild (n = 18) and moderate/severe (n = 29) stages of endometriosis. Data were assessed by the Wilcoxon rank-sum test. *P < 0.05. ns, no significance.

Supplementary Figure 3 Differential expression gene analysis of macrophage M2 and KEGG pathways and GO term enrichment analyses. **(A)** Volcano plot analysis of differentially expressed genes (DEGs) in M2 macrophages in the endometrium between normal and endometriosis tissues. **(B)** The heatmap of DEGs. **(C)** Dotplots of KEGG pathway enrichment analysis. **(D–F)** Dotplots of GO term enrichment analyses, including biological process **(D)**, cellular component **(E)**, and molecular function **(F)**.

## Abbreviations

EM, Endometriosis; GO, Gene Ontology; KEGG, Kyoto Encyclopedia of Genes and Genomes; DEGs, Differentially expressed genes; IHC, Immunohistochemistry; IF, Immunofluorescence; MCP-counter, Microenvironment Cell Populations-counter method; ImmuCellAI, Immune Cell Abundance Identifier.
