# Supplementary figures and images for: Identification and Validation of the Signatures of Infiltrating Immune Cells in the Eutopic Endometrium Endometria of Women With Endometriosis
Source: Front Immunol. 2021 Sep 3;12:671201. doi: 10.3389/fimmu.2021.671201 (PMC8446207; doi:10.3389/fimmu.2021.671201)

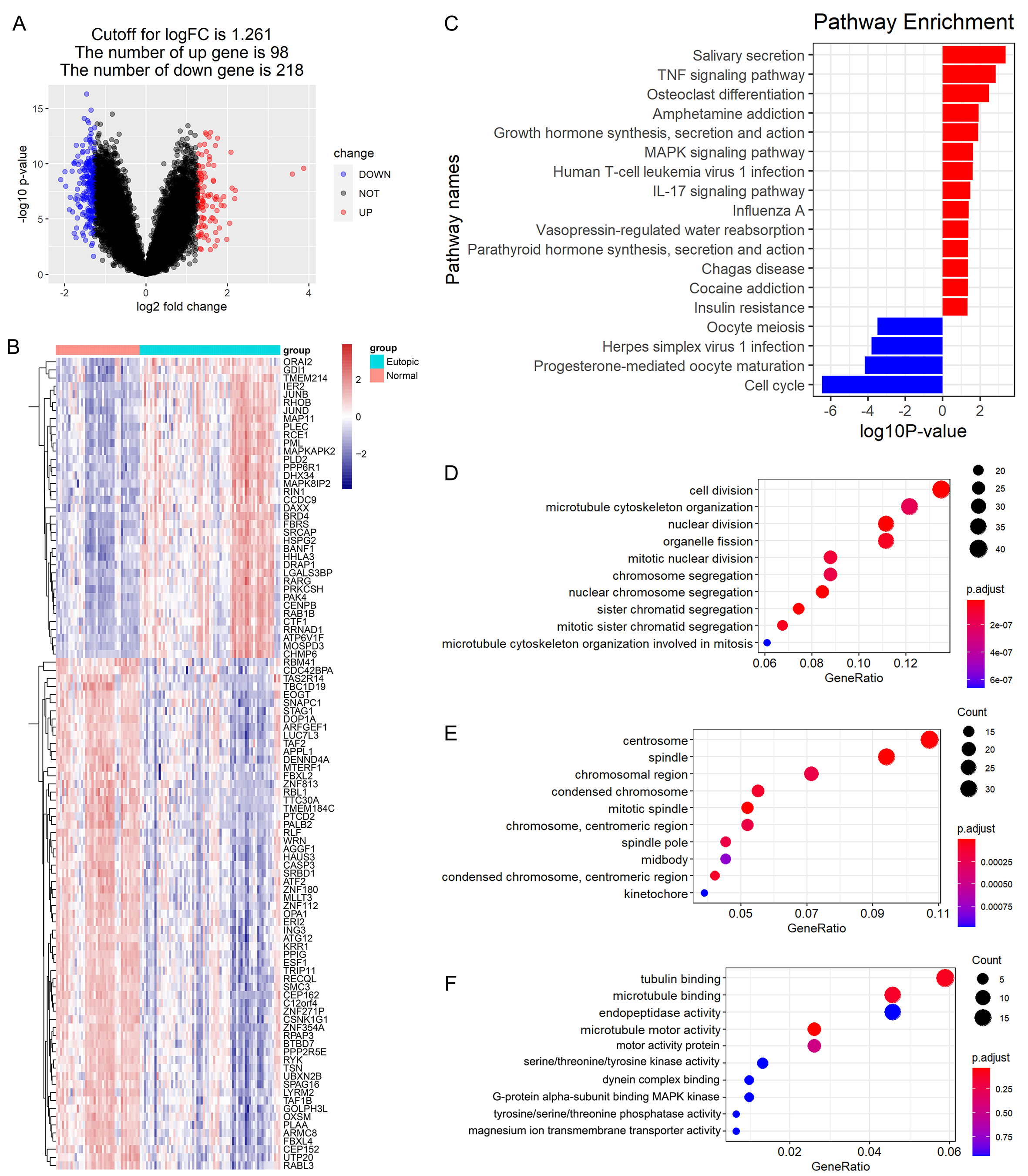

Supplement: Supplementary Figure 1 — Differential expression gene analysis of the endometrium between normal and endometriosis tissues and KEGG pathway and GO term enrichment analyses. (A) Volcano plot analysis of differentially expressed genes (DEGs) in the endometrium between normal and endometriosis tissues. (B) The heatmap of Top100 DEGs. (C) Bar plots of KEGG pathway enrichment analysis. (D–F) Dotplots of GO term enrichment analyses, including biological process (D), cellular component (E), and molecular function (F). [file Image_1.tif]

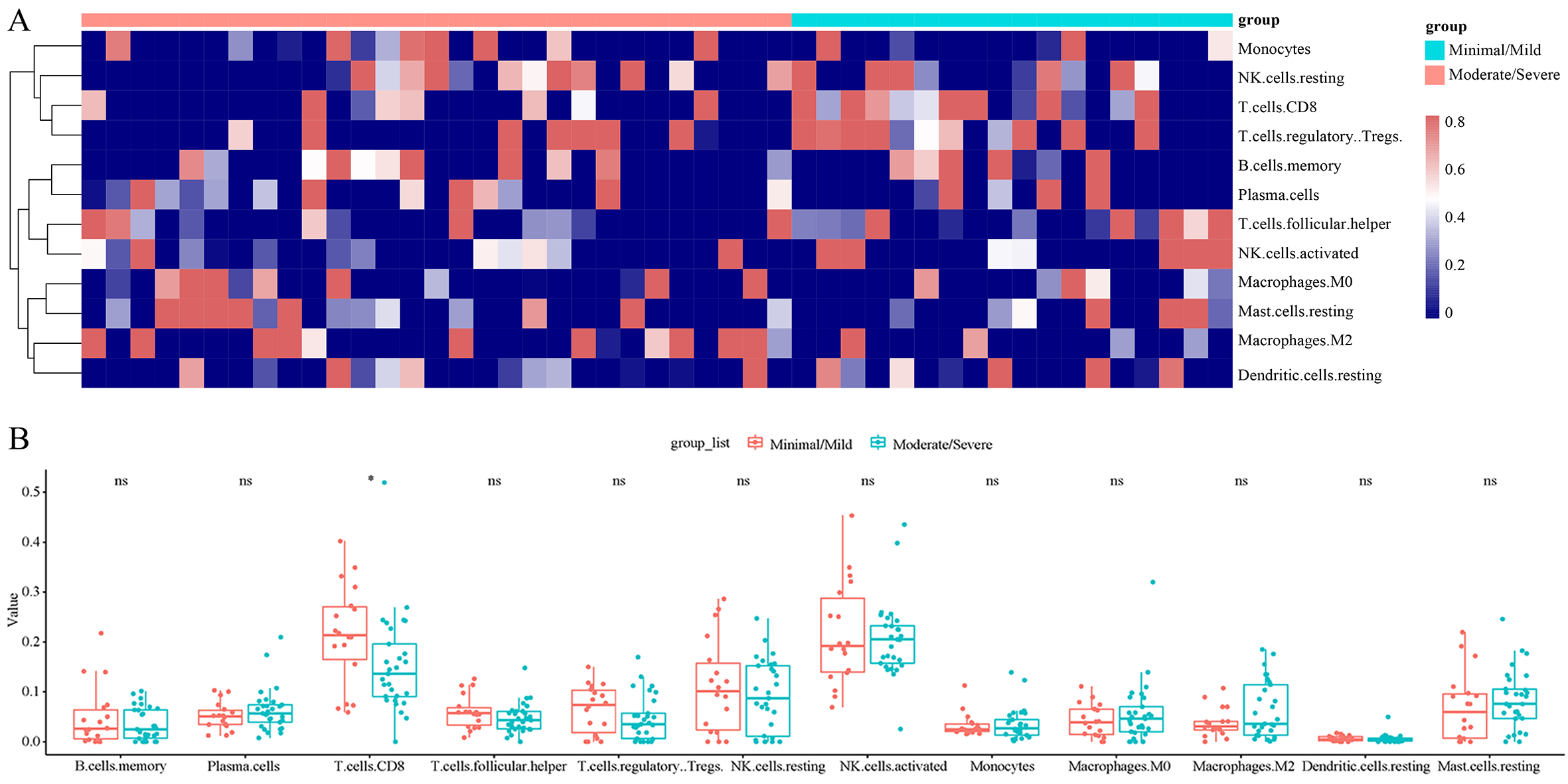

Supplement: Supplementary Figure 2 — Estimation of infiltrating immune cells in the endometria of ASRM clinical stages of endometriosis (GSE51981) by CIBERSORT. (A) The heatmap showing the estimated proportions of 12 major immune cell subsets in the endometria. (B) Boxplots comparing the proportions of 12 major immune cell subsets between minimal/mild (n = 18) and moderate/severe (n = 29) stages of endometriosis. Data were assessed by the Wilcoxon rank-sum test. *P < 0.05. ns, no significance. [file Image_2.tif]

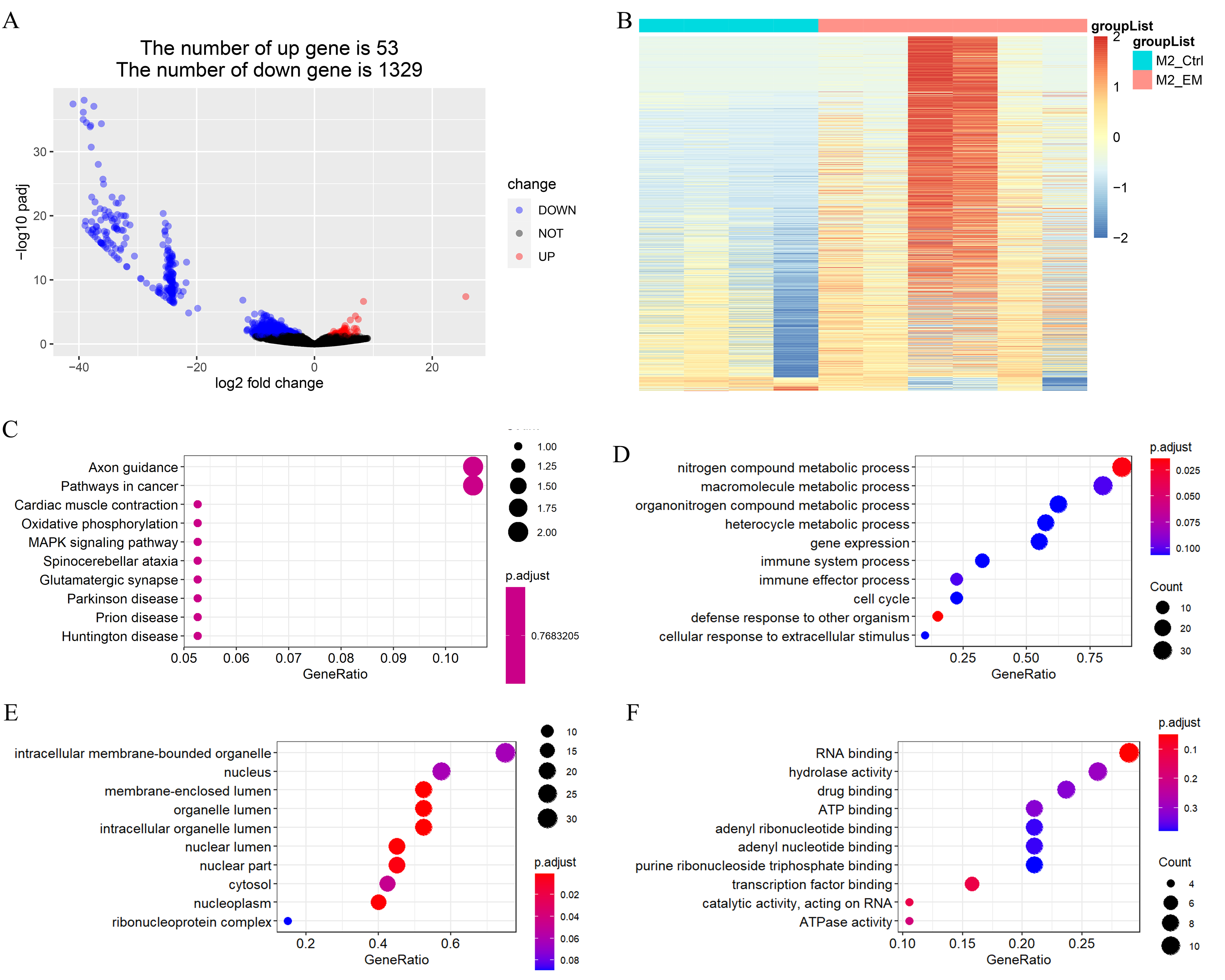

Supplement: Supplementary Figure 3 — Differential expression gene analysis of macrophage M2 and KEGG pathways and GO term enrichment analyses. (A) Volcano plot analysis of differentially expressed genes (DEGs) in M2 macrophages in the endometrium between normal and endometriosis tissues. (B) The heatmap of DEGs. (C) Dotplots of KEGG pathway enrichment analysis. (D–F) Dotplots of GO term enrichment analyses, including biological process (D), cellular component (E), and molecular function (F). [file Image_3.tif]
